# ATM is a key driver of NF-κB-dependent DNA-damage-induced senescence, stem cell dysfunction and aging

**DOI:** 10.18632/aging.102863

**Published:** 2020-03-22

**Authors:** Jing Zhao, Lei Zhang, Aiping Lu, Yingchao Han, Debora Colangelo, Christina Bukata, Alex Scibetta, Matthew J. Yousefzadeh, Xuesen Li, Aditi U. Gurkar, Sara J. McGowan, Luise Angelini, Ryan O’Kelly, Hongshuai Li, Lana Corbo, Tokio Sano, Heather Nick, Enrico Pola, Smitha P.S. Pilla, Warren C. Ladiges, Nam Vo, Johnny Huard, Laura J. Niedernhofer, Paul D. Robbins

**Affiliations:** 1 Department of Molecular Medicine and the Center on Aging, Scripps Research, Jupiter, FL 33458, USA; 2 Institute on the Biology of Aging and Metabolism and Department of Biochemistry, Molecular Biology and Biophysics, University of Minnesota Medical School, Minneapolis, MN 55415, USA; 3 Department of Immunology, University of Pittsburgh School of Medicine, Pittsburgh, PA 15261, USA; 4 Department of Orthopaedic Surgery, McGovern Medical School, University of Texas Health Science Center at Houston, Houston, TX 77030, USA; 5 Steadman Philippon Research Institute, Vail, CO 81657, USA; 6 Department of Orthopaedic Surgery, Catholic University of Rome School of Medicine, “A. Gemelli” University Hospital, Roma, Italy; 7 Department of Orthopaedic Surgery, University of Pittsburgh School of Medicine, Pittsburgh, PA 15261, USA; 8 Department of Comparative Medicine, University of Washington, Seattle, WA 98195, USA

**Keywords:** ATM, NF-κB, DNA damage response, cellular senescence, aging

## Abstract

NF-κB is a transcription factor activated in response to inflammatory, genotoxic and oxidative stress and important for driving senescence and aging. Ataxia-telangiectasia mutated (ATM) kinase, a core component of DNA damage response signaling, activates NF-κB in response to genotoxic and oxidative stress via post-translational modifications. Here we demonstrate that ATM is activated in senescent cells in culture and murine tissues from *Ercc1*-deficient mouse models of accelerated aging, as well as naturally aged mice. Genetic and pharmacologic inhibition of ATM reduced activation of NF-κB and markers of senescence and the senescence-associated secretory phenotype (SASP) in senescent *Ercc1^-/-^* MEFs. *Ercc1^-/Δ^* mice heterozygous for *Atm* have reduced NF-κB activity and cellular senescence, improved function of muscle-derived stem/progenetor cells (MDSPCs) and extended healthspan with reduced age-related pathology especially age-related bone and intervertebral disc pathologies. In addition, treatment of *Ercc1*^-/∆^ mice with the ATM inhibitor KU-55933 suppressed markers of senescence and SASP. Taken together, these results demonstrate that the ATM kinase is a major mediator of DNA damage-induced, NF-κB-mediated cellular senescence, stem cell dysfunction and aging and thus represents a therapeutic target to slow the progression of aging.

## INTRODUCTION

With aging there is an inevitable and progressive loss of the ability of tissues to recover from stress, leading to the increased incidence of chronic degenerative diseases. The loss of tissue homeostasis is driven, in part, by an increase in cellular senescence and a decline in stem cell function, resulting in various aging-related diseases, including osteoporosis, intervertebral disc degeneration, chronic kidney disease, diabetes, neurodegeneration and cancer [1–5]. Cellular senescence, characterized by irreversible cell cycle arrest with sustained metabolic activity, is a biological process that is physiologically required during embryonic development, wound healing and tumor suppression [6–10]. Importantly, senescent cells can develop a senescence-associated secretory phenotype (SASP), including expression of IL-6, IL-1α, IL-1β and TNF-α, that affects neighboring cells, disrupts stem cell niches, alters extracellular matrix, and induces secondary senescence [8, 11, 12]. Cellular senescence is mediated by p53/p21 and p16^INK4a^/retinoblastoma (Rb) tumor suppressor pathways in response to stress [8, 13] conferred by telomere attrition, DNA damage, oxidative and inflammatory stress and oncogene dysregulation [8].

Cellular senescence directly contributes to the progression of aging. For example, depletion of p16^INK4a^ positive cells in both progeroid and naturally aged transgenic mice expressing an inducible apoptotic transgene from the p16^INK4a^ promoter leads to an extension of healthspan [2, 3]. Similarly, clearance of senescent cells using senolytic agents extends healthspan, improves adult stem cell function and extends lifespan in mice [4, 5, 14–17].

The DNA damage response (DDR), essential for genome stability and organismal survival [18–21], is mediated through pathways that recruit multi-protein complexes to sites of DNA double-strand breaks (DSBs) or stalled replication forks. In particular, the MRE11-RAD50-NBS1 (MRN) complex is recruited to sites of DSBs to facilitate the recruitment, retention and activation of the ataxia-telangiectasia mutated (ATM) kinase [22]. Autophosphorylation of ATM at Ser1981 enhances its kinase activity, leading to phosphorylation of histone H2A variant H2AX (γH2AX) in nucleosomes surrounding damaged sites and recruiting more ATM and other repair factors [22, 23]. Additional DDR proteins, including KRAB-associated protein-1 (KAP1), p53 and checkpoint kinase 2 (CHK2), are phosphorylated by ATM kinase, promoting DNA repair, cell-cycle arrest, apoptosis and/or senescence [24, 25].

The Nuclear Factor κB (NF-κB) family of transcription factors consists of five members in mammalian cells, including RelA (p65), RelB, c-Rel, p50/p105 and p52/p100 [26]. All NF-κB subunits contain a Rel-homology domain (RHD), which is essential for DNA binding activity and dimerization. NF-κB functions to regulate innate and adaptive immune responses, embryonic development, proliferation, apoptosis, oncogenesis and senescence [26]. The canonical NF-κB pathway is activated by inflammatory stimuli, such as TNF-α, IL-1β and LPS, which lead to the activation of the IκB kinase (IKK). IKK is composed of two catalytic subunits, IKKα and IKKβ, and one regulatory subunit, NF-κB essential modifier (NEMO) or IKKγ [26]. Activated IKK complex phosphorylates the inhibitory protein IκBα to facilitate its polyubiquitination and degradation by the 26S proteosome, resulting in the translocation of the NF-κB heterodimer into the nucleus where it regulates gene transcription [26]. In addition, genotoxic stress activates a TNF-α-independent, but ATM-dependent NF-κB pathway via nuclear-localized NEMO [27, 28]. ATM phosphorylates NEMO at Ser85, which in turn induces sumoylation and mono-ubiquitination of NEMO at Lys277 and 309 [29]. These post-translational modifications eventually lead to the nuclear export of the ATM-NEMO complex to the cytoplasm where it associates with ubiquitin and SUMO-1 modified RIP1 and TAK1, activating the catalytic IKKβ subunit [28, 30].

NF-κB activity increases in multiple tissues of humans and rodents with aging and promotes cellular senescence [31–36]. Genetic depletion of RelA/p65 in aged mouse skin and a mouse model of human progeroid syndrome, reversed gene expression signature of aging and aging phenotypes [35, 37]. In addition, heterozygosity of p65/RelA in a mouse model of Hutchinson-Gilford Progeria Syndrome (*Zmpste24^-/-^*) resulted in attenuated aging pathology and a prolonged lifespan, linked in part to a reduced systemic inflammatory response and a reduction in ATM/NEMO-mediated NF-κB activation [34]. In addition, Nfkb1^-/-^ (p50^-/-^) mice have increased low-grade inflammation with signs of premature aging, including neural degeneration, impaired regeneration and declined overall lifespan [38–41]. Activation of NF-κB also is associated with multiple aging-related chronic diseases, including Alzheimer’s disease, Parkinson’s disease, Type II diabetes, osteoporosis and atherosclerosis [42], possibly through an increase in secretion of SASP factors [43].

DNA damage is known to increase with aging as demonstrated by an increase in DNA damage foci (γH2AX) and oxidative DNA lesions (8,5’-cyclopurines) [44, 45]. Intriguingly, persistent DDR signaling mediated by ATM activation has been reported to contribute to cellular senescence and SASP [46]. *In vitro*, SASP is dependent on ATM activation, suggesting a molecular link between ATM and NF-κB [8, 46, 47]. However, it is still unclear if aberrant DNA damage-induced activation of ATM *in vivo* exacerbates the cellular stress response to increase NF-κB, senescence, SASP and subsequently aging.

To address the role of ATM in driving NF-κB mediated senescence and aging, we used *Ercc1^-/Δ^* mice that model a human progeroid syndrome caused by impaired repair of DNA damage. The mice express only 5% of the normal level of the DNA repair endonuclease ERCC1-XPF that is required for nucleotide excision, interstrand crosslink and repair of some double-strand breaks. As a consequence, the *Ercc1^-/Δ^* mice spontaneously and rapidly develop progressive age-related diseases, including osteoporosis, sarcopenia, intervertebral disc degeneration, glomerulonephropathy, neurodegeneration, peripheral neuropathy and loss of cognition [48].

Here, we demonstrate that ATM and downstream effectors are persistently elevated in *Ercc1^-/∆^* and naturally aged mice, concomitant with hyperactive NF-κB signaling. Reducing ATM activity either genetically or pharmacologically reduced cellular senescence and downregulated NF-κB activation in cell culture. Importantly, *Ercc1^-/Δ^* mice heterozygous for *Atm* exhibited significantly reduced NF-κΒ activity, reduced cellular senescence, improved muscle-derived stem/progenitor cell function and attenuated age-related bone and intervertebral disc pathologies, leading to an extension of healthspan. Similarly, inhibiting ATM in *Ercc1*^-/∆^ mice by treatment with the ATM inhibitor KU-55933 reduced senescence and SASP marker expression. These results demonstrate a key role for ATM in aging and suggest that it is a therapeutic target for delaying or improving numerous age-related diseases.

## RESULTS

### NF-κB and ATM signaling are highly activated in cellular senescence, as well as accelerated and natural aging

**Figure 1:**
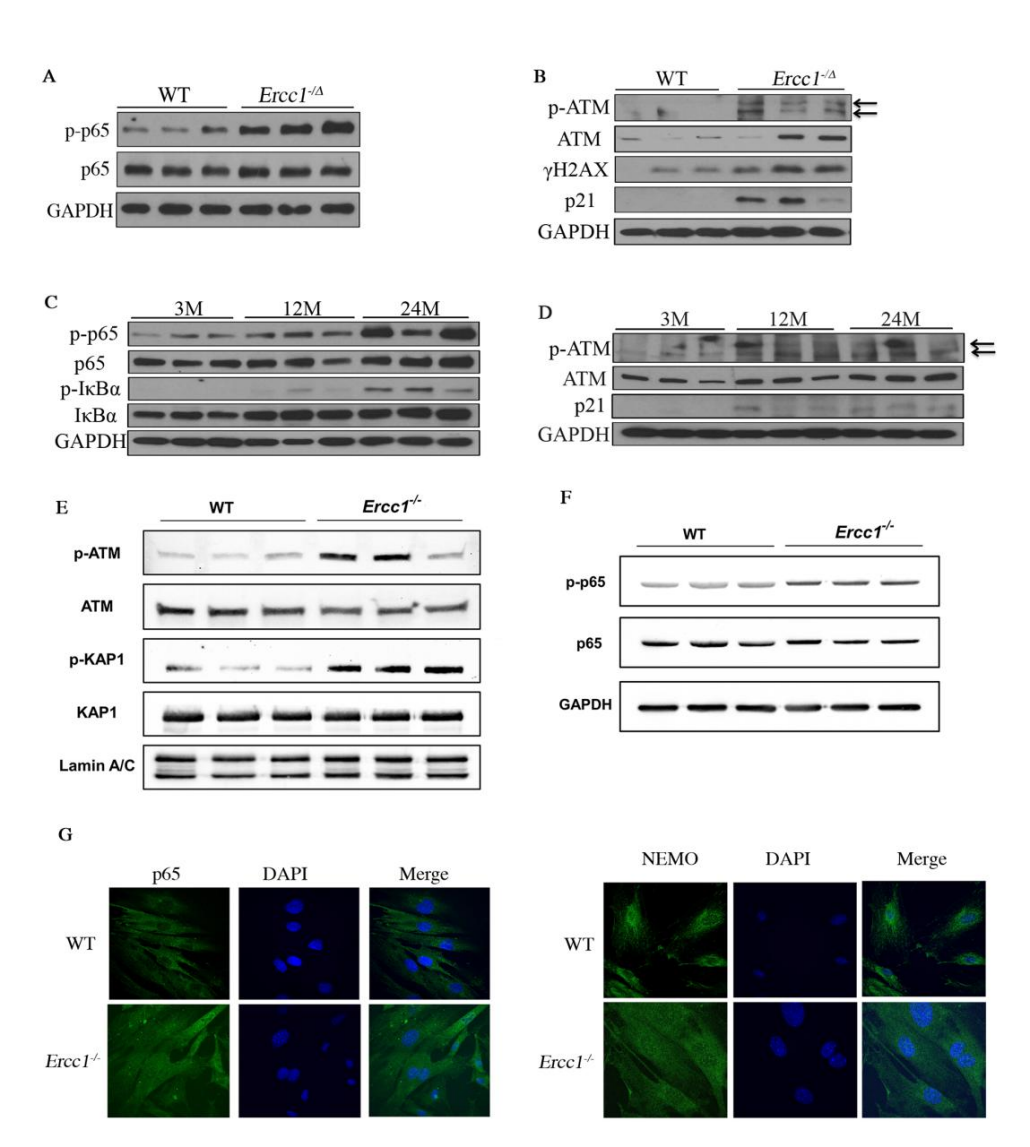
**DDR and NF-κB are activated concomitantly in senescent MEFs and aged tissues.** (**A**) Immunoblot detection of p-p65 and total p65 in liver tissue from 16-week-old WT (n=3) and *Ercc1^-/Δ^* (n=3) mice. (**B**) Immunoblot detection of phosphorylation of ATM and downstream targets γH2AX and p21 in liver from 16-week-old WT and *Ercc1^-/Δ^* mice. (**C**) Immunoblot detection of phosphorylation of NF-κB and IκBα in liver lysates from 3, 12 and 24 month-old WT mice. n=3 mice per group. (**D**) Immunoblot detection of p-ATM, ATM and p21 in the same liver lysates. (**E**) Immunoblot detection of DDR effectors in nuclear extracts from passage 5 WT and Ercc1^-/-^ MEFs, grown at 20% oxygen. (**F**) Level of NF-κB activation is higher in *Ercc1^-/-^* MEFs compared to WT MEFs at passage 5, as measured by Immunoblot detection of p-p65 and total p65 in WT and *Ercc1^-/-^* MEFs at passage 5 after culturing in 20% oxygen. (**G**) Representative images of immunofluorescent detection of p65 and NEMO in passage 4 WT and *Ercc1^-/-^* MEFs grown at 20% oxygen. Blue: DAPI staining; Green: p65 (top panel) or NEMO (bottom panel). Images were taken at the magnification of 60x.

Our previous studies using transgenic mice carrying a NF-κB-dependent EGFP reporter demonstrated an increase in the percentage of EGFP-positive cells in the liver, kidney, skeletal muscle and pancreas of progeroid *Ercc1^-/Δ^* and aged wild-type (WT) mice [35]. To further quantify NF-κB activation with aging, p-p65 (Ser536), a marker of NF-κB activation, was measured in murine liver (Figure 1A). Phosphorylation of p65 was significantly increased in 16-week-old *Ercc1^-/Δ^* mice compared to age-matched WT mice (Supplementary Figure 1A). In addition, there was an increase in the level of p-ATM as well as two senescence markers, γH2AX [49] and p21, in *Ercc1^-/∆^* liver compared to WT controls (Figure 1B and Supplementary Figure 1B). To determine if NF-κB and ATM were activated in WT mice with aging, p-p65, p-IκBα and p-ATM were measured by immunoblot in liver extracts from WT mice at multiple ages. The levels of p-p65 and p-IκBα increased gradually with age from 3 to 12 and 24-months of age (Figure 1C and Supplementary Figure 1C). These correlated with increased levels of p-ATM and the senescence marker p21 at 12 and 24 months of age (Figure 1D and Supplementary Figure 1D).

To examine ATM and NF-κΒ activation with senescence, the phosphorylation of ATM and NF-κΒ targets were measured initially in primary *Ercc1^-/-^* mouse embryonic fibroblasts (MEFs), which undergo premature senescence at 20% O_2_. The levels of p-ATM and p-KAP1 were increased in *Ercc1^-/-^* MEFs compared to WT MEFs (Figure 1E). Moreover, p-p65 levels were increased in passage 5 *Ercc1^-/-^* MEFs compared to WT cells (Figure 1F). There also was an increase in nuclear staining of p65 and NEMO in *Ercc1^-/-^* MEFs compared to WT cells, indicating NF-κB activation through a NEMO-dependent manner (Figure 1G). These findings suggest that NF-κB and ATM are co-activated in cells and tissues with higher levels of DNA damage-induced senescence.

### Pharmacologic inhibition of ATM rescues senescence caused by genotoxic stress via suppressing NEMO-dependent NF-κB activation

**Figure 2:**
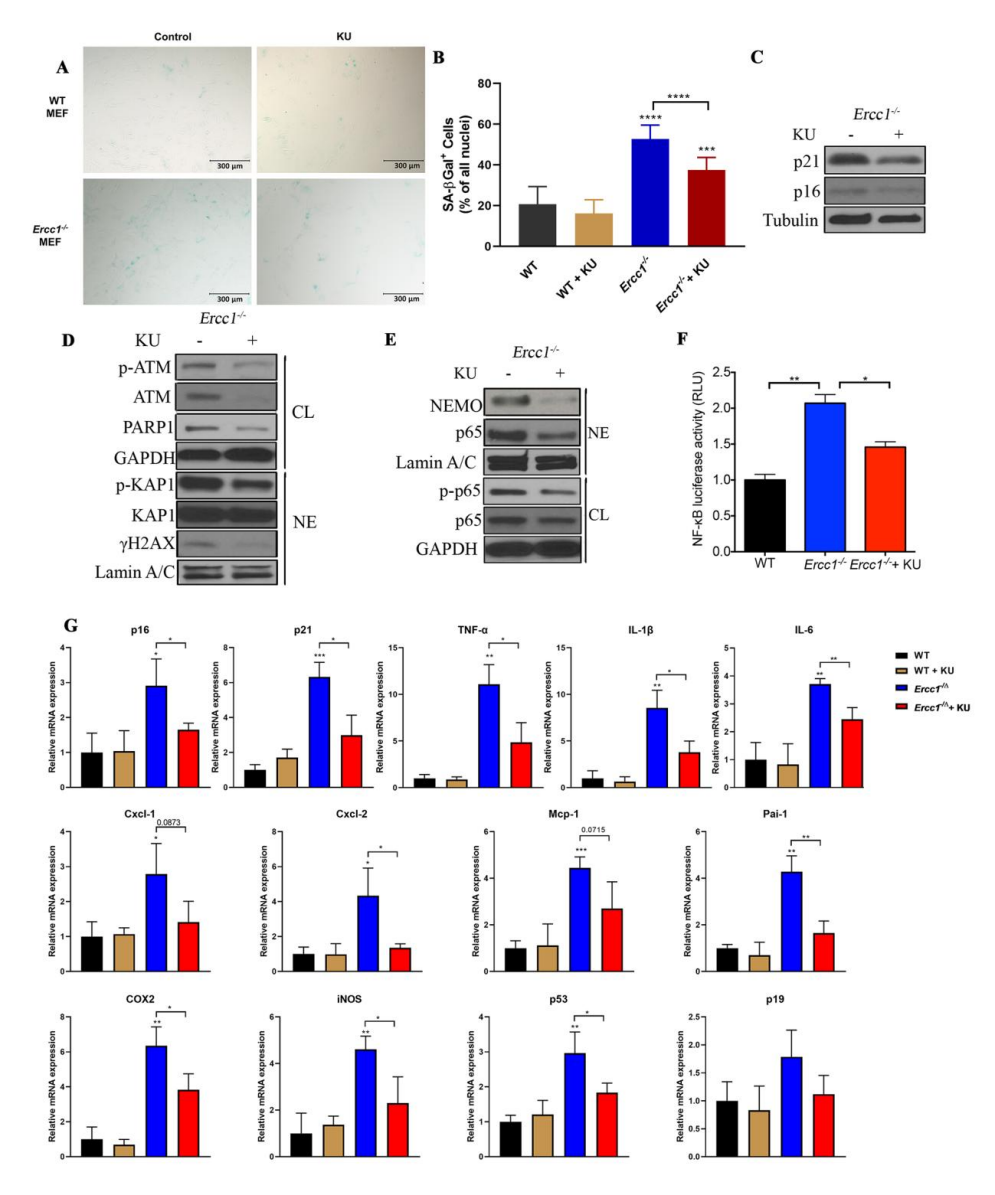
**Pharmacologic inhibition of ATM rescues oxidative stress-induced senescence by suppressing ATM- and NEMO-mediated NF-κB activation.** (**A**) Representative images of primary WT and *Ercc1^-/-^* MEFs were induced to undergo senescence by serial passaging at 20% oxygen. At passage 5, MEFs were grown in the presence or absence of KU-55933 (10 μM) for 72 hrs. Senescence was determined by SA-βgal staining. Images were obtained at the magnification of 10x. (**B**) Quantitation of the percent SA-βgal positive cells. Graph represents the mean +/- s.e.m. of three independent experiments. Student’s t-test, ***p <0.001, ****p <0.0001. (**C**) Passage 5 *Ercc1*^-/-^ MEFs treated with vehicle or KU-55933 (10 μM) for 72 hours were collected and levels of p21 and p16^INK4a^ were determined by western blotting. (**D**) Passage 5 *Ercc1^-/-^* MEFs were treated with KU-55933 (10 μM) for 72 hours and whole cell lysate (CL) and nuclear extracts (NE) were analyzed by immunoblotting for expression of proteins involved in the DNA damage response. (**E**) Whole cell lysate (CL) and nuclear extract (NE) were extracted from *Ercc1^-/-^* MEFs treated with 10 μM of KU-55933 for analysis of nuclear NEMO and p65. GAPDH was used as a loading control of total proteins and LaminA/C as a loading control of nuclear protein. (**F**) Passage 5 WT and *Ercc1^-/-^* MEFs transfected with a NF-κB-luciferase reporter construct were cultured in the presence or absence of KU-55933 (10 μM) and were collected for luciferase assays after 72 hours. (**G**) qRT-PCR analysis of mRNA expression in passage 5 WT and *Ercc1^-/-^* MEFs treated with or without of KU-55933 (10 μM) for 72 hrs. P values were determined using a Student’s t-test. *p<0.05, **p<0.01, ***p <0.001.

To elucidate further the causative role of activated ATM and DDR signaling in driving senescence, the effect of a selective ATM kinase inhibitor, KU-55933 [50], on senescence in MEFs was examined. Treatment of DNA repair deficient *Ercc1^-/-^* MEFs with KU-55933 (10 μM) reduced the percent of SA-βgal positive cells to a level similar to WT MEFs (Figure 2A and 2B). Additional markers of senescence, including the cell-cycle regulators p21^Cip1^ and p16^INK4A^, also were decreased by KU-55933 treatment (Figure 2C). As expected, autophosphorylation of ATM at Ser1981 was downregulated by the ATM inhibitor, as were the levels of p-KAP1 and γH2AX (Figure 2D). Interestingly, ATM inhibition also decreased Poly [ADP-ribose] polymerase 1 (PARP1) abundance (Figure 2D), an enzyme that promotes DNA repair and chromatin remodeling, utilizing NAD^+^ as a cofactor [51]. Interestingly, our results also suggest that inhibition of ATM activity may regulate ATM expression at protein level as indicated by reduced ATM level (Figure 2D). Furthermore, ATM inhibition reduced the abundance of nuclear-localized p65 and NEMO and the level of p-p65 (Figure 2E), as well as NF-κB transcriptional activity, measured using a NF-κB luciferase reporter assay (Figure 2F). Finally, treatment with the ATM inhibitor significantly reduced expression of multiple senescence and SASP markers as determined by qRT-PCR (Figure 2G). Taken together, these results suggest that ATM activation triggered by endogenous DNA damage plays a critical role in driving cellular senescence, SASP and NF-κB activation in a NEMO-dependent manner.

### Genetic depletion of Atm decreases genotoxic stress-induced cellular senescence

**Figure 3:**
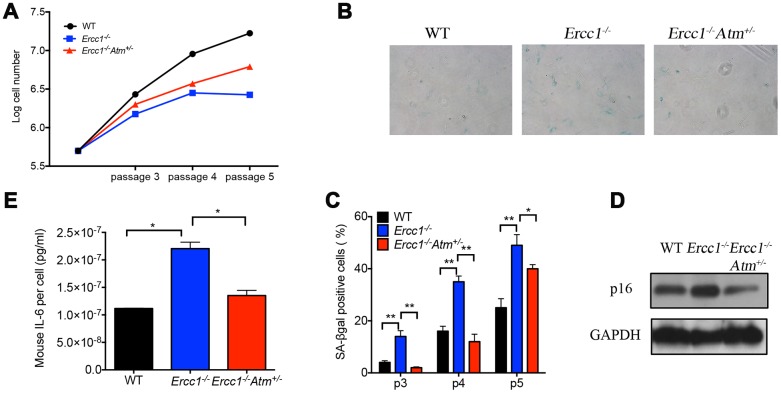
**Oxidative stress-induced cellular senescence is reduced by genetic depletion of *Atm.*** (**A**) Proliferation of WT (black), *Ercc1*^-/-^ (blue) and *Ercc1*^-/-^*Atm*^+/-^ (red) MEFs serially passaged at 20% oxygen was measured by an automated cell counter. Data shown are representative of three independent experiments using distinct MEF lines. (**B**) SA-βgal staining of serially passaged MEFs cultured at 20% oxygen. Shown are representative images of passage 5 WT, *Ercc1*^-/-^ and *Ercc1*^-/-^*Atm*^+/-^ MEFs taken at 10x magnification. (**C**) The average percentage of SA-βgal positive cells at each indicated passage. Ten fields were acquired and quantified per sample. Data shown are representative of two independent experiments. (**D**) Senescent WT, *Ercc1*^-/-^ and *Ercc1*^-/-^*Atm*^+/-^ MEFs (passage 5) cultured at 20% oxygen for 72 hrs were collected and lysed for immunoblot analysis of p16^INK4a^. (**E**) Supernatant collected from senescent WT, *Ercc1*^-/-^ and *Ercc1*^-/-^Atm^+/-^ MEFs was analyzed by ELISA for secreted IL-6. Graphs represent mean+/- s.e.m. P value was determined using Student’s t-test. *p<0.05, **p<0.01.

To eliminate possible off-target effects of the ATM inhibitor KU-55933, *Ercc1^-/-^* MEFs heterozygous for *Atm* (*Ercc1^-/-^Atm^+/-^*) were generated. The *Ercc1^-/-^Atm^+/-^* MEFs had increased proliferation compared to *Ercc1^-/-^* MEFs (Figure 3A). There also was a reduction in the percent of SA-ßgal^+^ *Ercc1^-/-^Atm^+/-^* MEFs compared to *Ercc1^-/-^* MEFs (Figure 3B and 3C). Expression of *p16^INK4a^* also was reduced in the double mutant MEFs (Figure 3D). Finally, there was a reduction in the level of secreted IL-6, a SASP factor, in conditioned media from *Ercc1^-/-^Atm^+/-^* MEFs compared to *Ercc1^-/-^* cells (Figure 3E). Taken together, these results suggest that ATM promotes genotoxic stress-induced cellular senescence, in part, through the activation NF-κB signaling.

### Genetic depletion of Atm extends healthspan in *Ercc1^-/Δ^* mice by reducing cellular senescence

**Figure 4:**
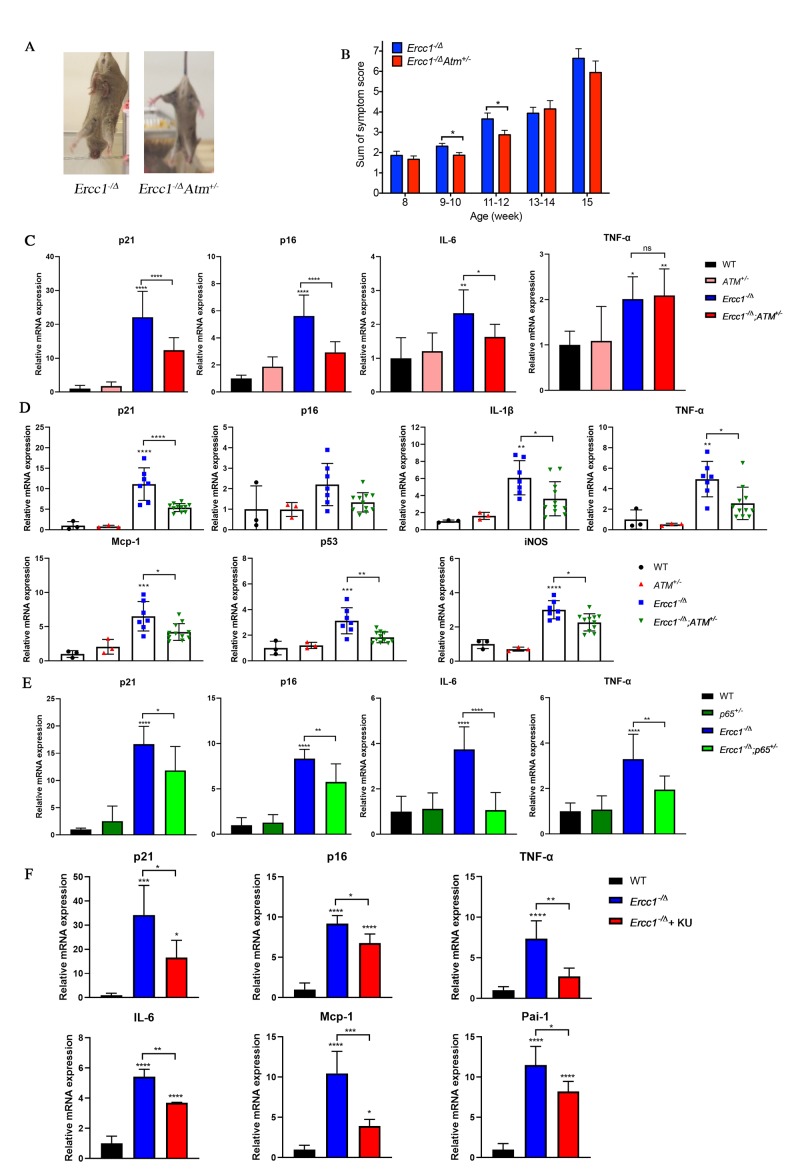
**Genetic reduction of *Atm* attenuates aging phenotypes and reduces cellular senescence *in vivo*.** (**A**) Representative images (left panel) of 15-week-old *Ercc1*^-/Δ^ and *Ercc1*^-/Δ^*Atm*^+/-^ mice illustrating the severity of their dystonia. (**B**) The composite score of aging symptoms (right panel) was plotted at the indicated ages. n=8-10 mice per group. (**C**) qRT-PCR analysis of mRNA expression in liver from 12-week-old WT, *Atm*^+/-^, *Ercc1*^-/Δ^ and *Ercc1*^-/Δ^*Atm*^+/-^ mice. n=3-6 per group. (**D**) qRT-PCR analysis of mRNA expression in quadriceps from 12-week-old WT, *Atm*^+/-^, *Ercc1*^-/Δ^ and *Ercc1*^-/Δ^*Atm*^+/-^ mice. n=3-11 per group. (**E**) qRT-PCR analysis of mRNA expression in liver from 10 to 12-week-old WT, *p65^+/-^*, *Ercc1*^-/Δ^ and *Ercc1*^-/Δ^p65^+/-^ mice. n=4-5 per group. (**F**) mRNA expression of senescence markers in the liver of 12-week-old *Ercc1*^-/Δ^ mice treated with 10 mg/kg of KU-55933 intraperitoneally 3 times per week for two weeks. n = 3 per group. Graphs represent mean+/- s.e.m. P value was determined using Student’s t-test. *p<0.05, **p<0.01, ***p <0.001, ****p <0.0001.

To determine if *Atm* heterozygosity extends healthspan in *Ercc1^-/∆^* mice, age-related symptoms, including kyphosis, tremor, ataxia, gait disorder, hind limb muscle wasting, forelimb grip strength and, in particular, dystonia were measured weekly in *Ercc1^-/∆^* and *Ercc1^-/∆^Atm^+/-^* mice. As shown in Figure 4A and 4B, *Atm* heterozygosity reduced the severity and slowed progression of aging symptoms in *Ercc1^-/Δ^* mice.

To determine if the extended healthspan correlated with reduced cellular senescence in *Ercc1^-/∆^Atm^+/-^* mice, the levels of expression of senescent markers and SASP factors were measured by qRT-PCR analysis. Expression of p21 was significantly reduced in 12-week-old *Ercc1^-/∆^Atm^+/-^* livers compared to those from aged-matched *Ercc1^-/∆^*mice, as was IL-6, a SASP factor and NF-κB target gene (Figure 4C). Similarly, the expression of senescence markers and SASP factors (except p16) were significantly reduced in *Ercc1^- /∆^Atm^+/-^* quadriceps compared with those in *Ercc1^-/∆^*mice (Figure 4D). In wildtype mice, there was no effect of ATM heterozygosity on expression of these senescence and SASP markers. Interestingly, IL-6 and TNF-α, but not p21^Cip1^ or p16^INK4a^ expression, were significantly lower in liver of 12-week-old *Ercc1^-/Δ^ p65^+/-^* mice compared to age-matched *Ercc1^-/Δ^Atm^+/-^* mice (Figure 4E). This indicates a distinct role for NF-κB in promoting aging. Taken together, these results suggest that genetic reduction of ATM leads to a reduction in cellular senescence and aging symptoms *in vivo*.

To determine if pharmacologic inhibition of ATM in *Ercc1*^-/∆^ mice confers a similar reduction in senescence and SASP markers as ATM heterozygosity, *Ercc1*^-/∆^ mice were treated three times per week i.p. for 2 weeks with 10 mg/kg of KU-55933. The mice were then analyzed for the extent of senescence and SASP in different tissues. As shown in Figure 4F, the levels of expression of senescent markers and SASP factors were measured were significantly reduced in liver tissues from *Ercc1^-/∆^*mice compared to untreated controls.

### Atm haploinsufficiency improves function of muscle-derived stem/progenitor cells (MDSPC)

**Figure 5:**
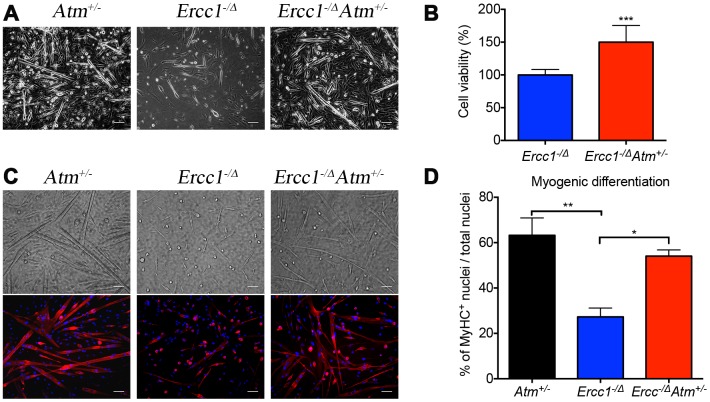
***Atm* heterozygosity improves muscle stem cell function and muscle regeneration.** Myogenic progenitor cells (myoblasts) and MDSPCs were isolated via preplate technique, 3 days after myoblasts were obtained, and the bright field pictures were taken. A total of three populations of *Atm*^+/-^, *Ercc1*^-/∆^*Atm*^+/-^ and *Ercc1*^-/∆^ were isolated from distinct mice and tested. All scale bars = 100 μm. (**A**) MDSPCs were cultured in myogenic differentiation medium for 3 days. Bright field images were taken and the cell fusion into multinucleated myotubes was determined by immuno-staining for MyHCf, a terminal myogenic differentiation marker. (**B**) Cell proliferation of MDSPCs was measured using an MTS assay. The graph displays the average of three populations. Error bars indicate mean ± SD. ***P<0.001 (**C**) Representative images of immunofluorescence detection of differentiated myofibers. All scale bars in panel C=50 μm. (**D**) Myogenic differentiation was quantified by determining the number of nuclei in MyHCf positive myotubes relative to the total number of nuclei in the culture. Error bars indicate “mean ±SD”. *P<0.05. **P<0.01. Error bars indicate “mean ± SD”. *P<0.05. Two-tailed Student’s t-test was performed.

A decline in stem cell function has long been associated with aging, which in the musculoskeletal system leads to sarcopenia and muscle wasting [1, 52]. We previously reported that muscle-derived stem/progenitor cells (MDSPCs) isolated from *Ercc1^-/Δ^* mice failed to proliferate or differentiate properly, similar to MDSCPs from naturally aged mice [53]. Interestingly, the function of MDSPCs isolated from *Ercc1^-/∆^* mice was partially restored by *Atm* heterozygosity (Figure 5). In fact, MDSPCs isolated from *Ercc1^-/∆^Atm^+/-^* mice showed similar levels of myogenic differentiation to WT mice (Figure 5A, 5C and 5D). Proliferation of MDSPCs isolated from *Ercc1^-/∆^Atm^+/-^* mice also increased by 50% compared to control MDSPCs (Figure 5B). This result is consistent with delayed onset and reduced severity of gait disorder observed in *Ercc1^-/Δ^Atm^+/-^* mice, which is partly due to severe muscle wasting (data not shown).

### Atm haploinsufficiency improves aging-related pathology in certain tissues

**Figure 6:**
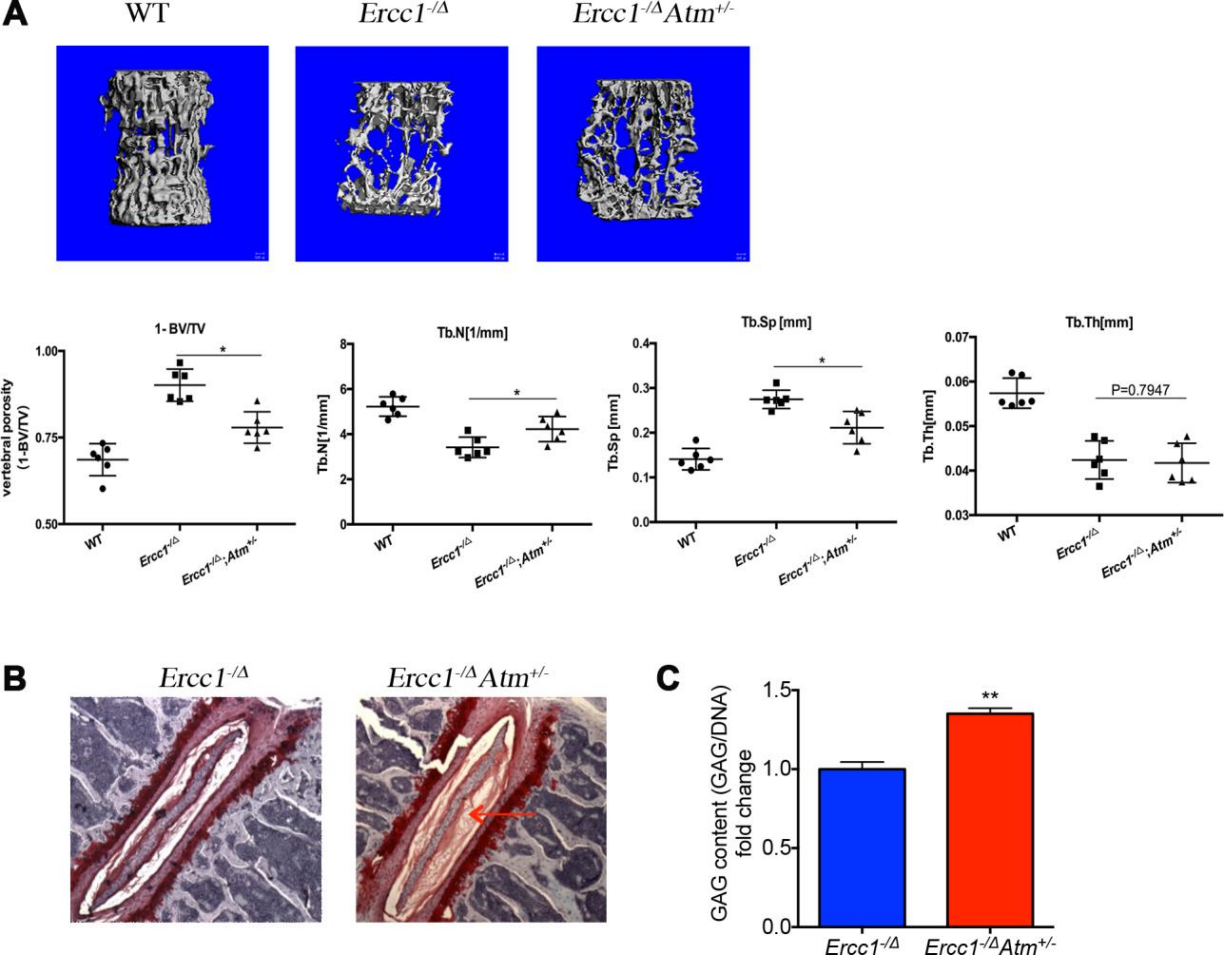
**Genetic reduction of *Atm* improves bone and intervertebral disc pathology in progeroid *Ercc1*^-/Δ^ mice.** (**A**) Representative micro-CT images of lumber spines comparing severity of osteoporosis in 16-week-old WT, *Ercc1*^-/Δ^, *Ercc1*^-/Δ^*Atm*^+/-^ mice. n=3-5 per group. Quantification of vertebral porosity, trabecular number, trabecular separation, thickness of trabecular bone was performed and shown. (**B**) Safranin O staining for disc matrix in thoracic discs from 12-week-old *Ercc1*^-/Δ^ and *Ercc1*^-/Δ^*Atm*^+/-^ mice. (**C**) GAG content measured by DMMB assays with NP tissues isolated from 12-week-old lumber discs. n=3 each group. Mean+/- s.e.m. P value was determined using Student’s t-test. **p<0.01.

To further assess the effect of ATM status on aging, tissues from 12-week *Ercc1^-/∆^* and *Ercc1^-/∆^Atm^+/-^* mice were examined histologically. There are reduced lymphoid aggregates in the liver and kidney of *Ercc1^-/∆^* and *Ercc1^-/∆^Atm^+/-^* mice, suggesting reduced inflammatory infiltration in tissues at the age analyzed [54]. In addition, *Ercc1^-/∆^Atm^+/-^* mice had reduced age-related bony changes in the lumbar vertebrae, as determined using μCT. Compared to WT mice, *Ercc1^−/∆^* mice showed marked trabecular bone loss (Figure 6A), signified by an increase in osteoporosis (1-BV/TV), a decrease in trabecular number (Tb.N) and trabecular thickness (Tb.Th) and an increase in the trabecular spacing (Tb.Sp). *Atm* heterozygosity significantly improved bone qualities when compared with *Ercc1^−/∆^* mice, showing reduced vertebral osteoporosis and trabecular spacing, accompanied by a significant increase in trabecular number (Figure 6A). However, no significant difference was found in trabecular thickness (Figure 6A). *Ercc1^-/∆^Atm^+/-^* mice also had improved Safranin O staining of the intervertebral disc from the lumbar spines, consistent with significantly higher levels of glycosaminoglycans (GAGs) in the nucleus pulposus (Figure 6B and 6C) [55–57]. GAG measurement indicates the level of the matrix proteoglycans, which plays a critical role in counteracting mechanical forces imparted on the spine [58]. Taken together, these results suggest that heterozygosity of *Atm* improves certain age-related conditions, in particular in the musculoskeletal system.

### Atm haploinsufficiency reduces NF-κB signaling in Ercc1^-/Δ^ mice

**Figure 7:**
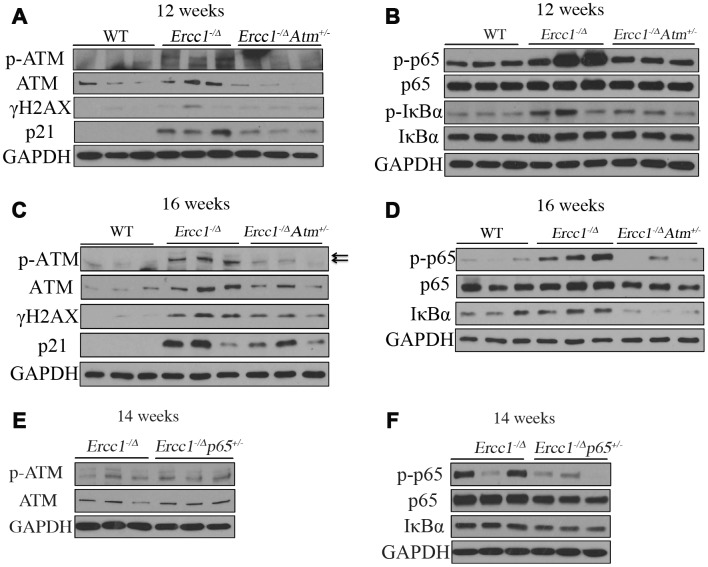
**ATM and NF-κB activation are downregulated in *Ercc1*^-/Δ^ mice heterozygous for *Atm*.** (**A**) Livers were collected at 12 weeks of age from WT, *Ercc1*^-/Δ^ and *Ercc1*^-/Δ^*Atm*^+/-^ mic (n=3 per genotype) and lysates analyzed by western blot for activation of ATM and its downstream effectors. (**B**) Same liver lysates were used to measure phosphorylation of p65 and IκBα. (**C**) Western blot analysis of livers from 16-week-old WT, *Ercc1*^-/Δ^ and *Ercc1*^-/Δ^Atm^+/-^mice (n=3 per genotype) probed for activation of ATM. GAPDH was used as a loading control. (**D**) Same liver lysates used to measure activation of NF-κB. (**E**) Fourteen-week-old livers from *Ercc1*^-/Δ^ and *Ercc1*^-/Δ^p65^+/-^ mice (n=3 per genotype) were analyzed by western blot for activation of ATM (**F**) and NF-κB.

To determine the extent to which ATM signaling mediates NF-κB activation *in vivo*, livers from 12- and 16-week-old *Ercc1^-/Δ^* and *Ercc1^-/∆^Atm^+/-^* mice were examined. There was a significant decrease in DDR signaling in *Ercc1^-/∆^Atm^+/-^* mice compared to *Ercc1^-/Δ^* mice as indicated by reduced levels of p-ATM and γH2AX (Figure 7A, 7C and Supplementary Figure 2A, 2C). Moreover, the levels of p21^Cip1^ were significantly decreased in livers from *Ercc1^-/∆^Atm^+/-^* mice, particularly at 12 weeks of age, consistent with reduced cellular senescence and the qRT-PCR results (Figure 4C). Importantly, the level of phospho-p65 was greatly reduced in *Ercc1^-/∆^Atm^+/-^* livers (Figure 7B, 7D and Supplementary Figure 2B, 2D), suggesting that NF-κB activation in *Ercc1^-/Δ^* mice is driven, at least in part, by ATM activation. Accordingly, there was reduced p-IκBα and IκBα levels in *Ercc1^-/∆^Atm^+/-^* mice compared to *Ercc1^-/Δ^* controls (Figure 7B, 7D and Supplementary Figure 2B, 2D) [59, 60].

To determine if the molecular changes in *Ercc1^-/∆^Atm^+/-^* mice mirror that in *Ercc1^-/Δ^p65^+/-^* mice, ATM and NF-κB activation was measured in *Ercc1^-/Δ^* and *Ercc1^-/Δ^p65^+/-^* mice. There was no difference in the level of ATM or p-ATM between *Ercc1^-/Δ^p65^+/-^* and *Ercc1^-/Δ^* mice (Figure 7E and Supplementary Figure 2E), suggesting that DDR/ATM signaling is the upstream regulator of NF-κB. The levels of p-p65 and total IκBα were reduced in *Ercc1^-/Δ^p65^+/-^* mice compared to *Ercc1^-/Δ^* mice (Figure 7F and Supplementary Figure 2F), consistent with qRT-PCR results (Figure 4E) showing reduced NF-κB-mediated transcription. Taken together, these results suggest that DDR signaling plays an essential role in NF-κB activation in response to endogenous DNA damage. Moreover, the deletion of one *Atm* allele is sufficient to attenuate DDR signaling and dampen NF-κB activation *in vivo*.

## DISCUSSION

**Figure 8:**
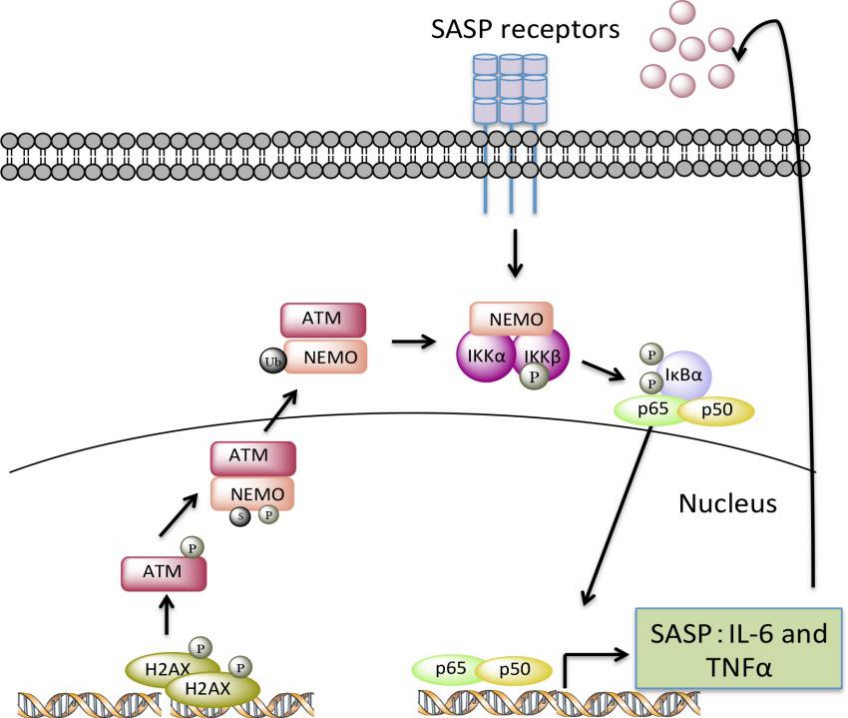
**A model depicting how endogenous nuclear DNA damage activates NF-κB via an ATM- and NEMO-dependent mechanism to drive cellular senescence and senescence-associated secretory phenotype (SASP).** In response to chronic accumulation of endogenous DNA damage, ATM undergoes autophosphorylation and promotes phosphorylation, SUMOylation, and monoubiquitylation of NEMO. As a result, monoubiquitylated NEMO along with ATM translocates to the cytoplasm, activating the IKK complex. Phosphorylation of IκB leads to the release of p65 so that it can translocate into nucleus upregulating a transcriptional program of certain SASP factors, such as TNFα and IL-6. Secreted SASP factors then trigger a second wave of NF-κB activation through cytokine receptors, further enhancing cell-autonomous pathway-mediated senescence and inducing non-cell-autonomous pathway-mediated senescence.

DNA damage is a critical factor in driving cellular senescence and aging [61]. Multiple human diseases of accelerated aging, such as XFE progeroid syndrome, Cockayne syndrome, Hutchinson-Gilford progeria and Werner syndrome are caused by inherited defects in maintaining genome integrity [48, 62]. Levels of oxidative DNA damage are increased in old or progeroid organisms compared to young [44, 63, 64]. Although the ATM kinase is a critical mediator of the DNA damage response, *in vivo* evidence linking chronic activation of ATM to senescence and aging is still lacking. Here, we demonstrate that ATM activation increases with aging in mammals. In addition, in the *Ercc1*^-/∆^ progeroid mouse model of accelerated aging, genetic reduction of ATM reduces cellular senescence, improves stem cell function, extends healthspan and reduces certain age-related pathologies in the musculoskeletal system. Moreover, we demonstrate that ATM promotes senescence and aging, at least in part, by regulating the transcriptional activity of NF-κB (Figure 8).

ATM activity is increased in liver with aging in not only progeroid *Ercc1^-/Δ^* mice, but also naturally aged WT mice. Similarly, there was an increase in NF-κB activity in livers that correlates with ATM activity. Conversely, a reduction in ATM activation *in vivo* either genetically or pharmacologically resulted in a reduction in the levels of γH2AX in liver and decreased expression of senescent markers and SASP, in particular *p21^Cip1^* and *Il6* (Figure 4C and 7A, 7C). Consistent with these observations, there was an increase in ATM and NF-κB activity in ERCC1-deficient cells grown under oxidative stress conditions in cell culture. Reduction of ATM either genetically or pharmacologically in MEFs also resulted in a reduction in oxidative stress-induced senescence along with reduction in NF-κB activation.

We previously demonstrated that heterozygosity in p65/RelA (*Ercc1^-/Δ^p65^+/-^*) in *Ercc1^-/Δ^* mice resulted in reduced senescence in multiple tissues as well as extended healthspan. Here we demonstrate that *Atm* heterozygosity reduced NF-κB activation to an extent similar to p65 heterozygosity in *Ercc1^-/Δ^* mice, suggesting that ATM kinase is a major activator of NF-κB in the context of DNA-damage mediated senescence and aging. As a result, expression of SASP factors transcriptionally regulated by NF-κB, especially IL-6, was down-regulated in livers of both *Ercc1^-/Δ^p65^+/-^* and *Ercc1^-/∆^Atm^+/-^* mice. These findings support previous studies reporting that ATM activation is indispensable for the SASP phenotype secreting inflammatory cytokines [46, 65]. Interestingly, p65/RelA heterozygosity resulted in a stronger reduction in IL-6 and TNF-α expression compared to *Atm* heterozygosity, suggesting either an ATM-independent pathway or that heterozygosity of *Atm* has less of an effect on the pathway. We speculate that DSBs activate NF-κB primarily through an ATM/NEMO-dependent pathway, which then increases the production of TNF-α and other SASP factors. These SASP factors, in turn, trigger a second wave of NF-κB activation, enhancing cell-autonomous and cell-non-autonomous senescence [30, 66] (Figure 8). Thus *Atm* haploinsufficiency only dampens the primary activation of NF-κΒ, while p65 heterozygosity targets both primary and secondary NF-κB activation, conferring a stronger inhibition of the SASP phenotype. These results also are consistent with a greater effect of heterozygosity in p65/RelA on liver and kidney pathology than *Atm* heterozygosity.

We previously reported that reduction in p65/RelA in muscle derived stem/progenitor cells (MDSPCs) from *Ercc1^-/∆^* mice improved their ability to proliferate as well as differentiate. Here we demonstrated that reduction in ATM activity also improved self-renewal and differentiation in muscle derived stem/progenitor cells, suggesting persistent activation of ATM-dependent signaling negatively regulates stem cell function (Figure 5). This consistent with the previous observation that depletion of p21^Cip1^, which is regulated by ATM-p53, restores stem cell self-renewal and tissue homeostasis without accelerating carcinogenesis in mice deficient in telomerase [67].

Moreover, we and others previously demonstrated that genetic and pharmacological reduction in p65/RelA improves bone architecture and reduces osteoporosis [34, 35]. Here we demonstrated that *Atm* haploinsufficiency also leads to significantly improved osteoporosis and reduced disc degeneration (Figure 6), suggesting ATM activation could contribute to increased chronic inflammation in spines via activating NF-κB in aging.

Persistent DNA damage-mediated DDR signaling, in particular ATM activation, has been demonstrated to be essential for the establishment of an NF-κB-dependent SASP phenotype in cell culture. Nuclear translocation of NEMO plays a critical role in relaying nuclear signal to cytoplasm to activate NF-κB in response to genotoxic stress [28]. For example, increased ATM phosphorylation and nuclear-localized NEMO were found in the *Zmpste24^-/-^* mouse model of Hutchinson-Gilford progeria syndrome (HGPS), where accumulation of nuclear prelamin-A leads to a perturbation of NF-κB signaling [34]. The transcription factor GATA4 was identified as a key mediator connecting DDR signaling to NF-κB activation and the senescent SASP phenotype [13]. In addition, ATM/IFI16 mediated non-canonical activation of DNA sensing adaptor STING was demonstrated recently in etoposide-induced nuclear DNA damage, leading to increased NF-κB activation [68]. However, in this study, we observed increased nuclear localization of NEMO in response to nuclear DNA damage, which could be ablated by an ATM inhibitor. Thus, our results support the presence of ATM/NEMO-dependent NF-κB pathway in response to chronic DNA damage.

ATM kinase is best known for its causal role in ataxia telangiectasia (AT), a rare autosomal recessive disease characterized by progressive neurodegeneration, immunodeficiency, cancer predisposition, radiosensitivity and premature skin aging [69–71]. Despite the fact that *Atm*-null mice have reduced dopaminergic neurons with age, decreased synaptic function in hippocampal neurons and defects in neuronal network activity, mice heterozygous for *Atm* improved not only neurodegenerative pathology, but also Huntington-like behavior in a mouse model of Huntington’s disease [22, 72, 73]. In addition, genetic and/or pharmacologic reduction of ATM reduced doxorubicin-induced cardiotoxicity and rescued cardiac inflammation and heart failure caused by DNA single-strand breaks [74, 75]. These observations are consistent with our results showing neurologic symptoms and musculoskeletal pathology were improved in *Atm* haploinsufficient *Ercc1^-/∆^* mice. In addition, our study showed that a short-term administration of KU55933 reduced multiple senescence and SASP markers in liver tissues in *Ercc1^-/∆^* mice. These results indicate that ATM kinase may be a potential drug target for the treatment of multiple aging-related diseases, especially those that have strong correlation with DNA damage accumulation. Given that heterozygous carriers of AT are symptom-free in general, we speculate that one WT allele of Atm is sufficient to exert its function in DNA repair and thereby doesn’t trigger the neurological degeneration as seen in AT patients. Interestingly, studies in Chinese and Italian nonagenarians/centenarians have identified a single nucleotide polymorphism (SNP, rs189037) of in the promoter region of ATM that moderately represses transcription of *Atm* by regulating its binding to an activator protein 2α (AP-2^α^). These results suggest that, similar to our results in *Ercc1^-/∆^* mice, a slight reduction in ATM can contribute to extended lifespan in humans [76, 77].

Taken together, our results suggest that ATM acts as the main stimulus of NF-κB activation in DNA damaged-induced senescence and aging. Reduction in ATM either genetically or pharmacologically is able to reduce the adverse effects of chronic DNA damage, reducing cellular senescence, improving stem cell function and extending healthpsan. Thus, ATM and NF-κB represent therapeutic targets, at least later in life, for improving frailty and certain aging-related diseases.

## MATERIALS AND METHODS

### Cells and mice

Primary mouse embryonic fibroblasts (MEFs) were isolated on embryonic day 12.5-13.5. In brief, mouse embryos were isolated from yolk sac followed by removal of viscera, lung and heart. Embryos were then minced into fine chunks, covered with media, cultured at 3% oxygen to reduce stresses and serially passaged. MEFs were grown in 1:1 of Dulbecco’s Modification of Eagles Medium (with 4.5 g/L glucose and L-glutamine) and Ham’s F10 medium, supplemented with 10% fetal bovine serum, penicillin and streptomycin and non-essential amino acids. To induce oxidative stress and oxidative DNA damage, MEFs were switched to 20% oxygen at passage 3.

*Ercc1^+/-^* and *Ercc1^+/Δ^* mice from C57BL/6J and FVB/NJ backgrounds were crossed to generate *Ercc1^-/Δ^* F1 hybrid mice. *Atm^+/-^* mice were crossed to *Ercc1^+/-^* from C57BL/6J background to generate *Ercc1^+/-^Atm^+/-^* mice, which were then bred with *Ercc1^+/Δ^* mice from FVB/NJ background to generate F1 *Ercc1^-/∆^Atm^+/-^* mice. Breeders were backcrossed for ten generations yielding F1 mice that are genetically identical. Animal protocols used in this study were approved by Scripps Florida Institutional Animal Care and Use Committee.

12-week-old *Ercc1*^-/Δ^ mice were injected intraperitoneally with 10 mg/kg of KU-55933 (AdooQ Bioscience, Irvine, CA, USA) or the same volume of vehicle (1% DMSO: 30% PEG400: 69% H_2_O) 3 times per week for two weeks. All mice were euthanized 1 hour after the last injection.

### Immunoblotting

MEFs were treated with either KU-55933 (10 μM, Selleckchem) or vehicle for 72 hours prior to harvesting for analysis of protein expression. Cell lysates were prepared with RIPA buffer (20 mM Tris-HCl (pH 7.5), 150 mM NaCl, 1 mM Na_2_EDTA, 1 μM EGTA, 1% NP-40, 1% sodium deoxycholate, 2.5 μM sodium pyrophosphate, 1 μM β-glycerophosphate, 1 μM Na_3_VO_4_ and 1 μg/ml leupeptin), supplemented with 1X of protease inhibitor cocktails (Sigma) and Halt phosphatase inhibitor cocktail (Thermo #78420). Snap-frozen liver and kidney samples were homogenized using FastPrep-24 instrument in RIPA buffer. Equal amounts of proteins (40 μg) were resolved on 4-15% Mini-PROTEAN TGX precast protein gels (Bio-Rad). Primary antibodies used are as follows: p-ATM Ser 1981 (cell lysates: Rockland cat. no. 200-301-400; tissue lysates: Santa Cruz sc-47739), ATM (CST #2873), p-KAP1 Ser 824(Abcam ab70369), KAP1 (Abcam ab22553), γ-H2AX (Novus NB100-384), PARP1 (CST #9532), p-p65 Ser 536 (CST #3033), p65 (CST #8242), p-IκBα Ser 32/36 (CST #9246), IκBα (Santa Cruz sc-371), p16^INK4a^ (Santa Cruz sc-1207), p21^Cip1^ (Santa Cruz sc-6246), LaminA/C (Santa Cruz sc-20681), anti-tubulin (CST #2146), and GAPDH (CST #5174). Blots were exposed to X-ray film in a dark room or developed on iBright™ FL1000 Imaging System (Figure 1E and 1F). Protein levels were quantified by Image J software.

### Nuclear extraction

Extraction of cytoplasmic and nuclear fractions was performed using the NE-PER nuclear and cytoplasmic extraction reagents (Thermo Fisher) according to the manufacturer’s instructions. Briefly, 1x10^6^ cells were suspended and lysed in CERI and CERII reagents consecutively on ice to obtain cytoplasmic fractions. Pellets of intact nuclei were then suspended in NER reagent to release nuclear contents.

### NF-κB luciferase reporter assay

Primary MEFs transfected with a NF-κB luciferase reporter construct were cultured in 6-well plates in triplicate in the absence or presence of KU-55933 (10 μM) for 72 hrs. Cells were then collected with Passive Lysis Buffer (Promega) and luciferase assay (Promega) was performed by using a luminometer according to the manufacturer’s instructions.

### Cell proliferation assay

Passage 3 MEFs were seeded at 5x10^5^ cells in 10-cm plates, allowed to grow for 72-96 hours to reach confluence at 20% oxygen and then trypsinized for determination of cell number. Serial passage was carried until passage 5 and cell number was determined for each passage. Cell number was measured using a Moxi Z Mini automated cell counter. Log cell number was plotted versus passage number.

### Enzyme-linked immunosorbent assay (ELISA)

Supernatant collected at the end of passage 4 from primary MEFs was analyzed for IL-6 production by ELISA using a mouse IL-6 ELISA kit (Becton Dickinson) according to the manufacturer’s instructions.

### Quantitative reverse transcription- polymerase chain reaction (qRT-PCR)

Snap-frozen tissues were preserved in RNAlater stabilization solution (Thermo Fisher). Total RNA was extracted using TRIZOL reagent (Life Technologies) and 1.5 μg of RNA was subjected to complementary DNA (cDNA) synthesis using SuperScript VILO cDNA synthesis kit (Thermo Fisher). qRT-PCR was performed with Platinum SYBR Green qPCR SuperMix-UDG with ROX (Thermo Fisher) in a StepOnePlus Real-Time PCR system. Relative expression of target genes was calculated using the comparative C_T_ method (ΔΔC_T_). ΔC_T_ was calculated by normalizing to an internal control gene *Actb* (β-actin) and ΔΔC_T_ by normalizing to the mean ΔC_T_ value of the control group. Primers used are as follows: *Cdkn1a* (p21^Cip1^) forward: GTCAGGCTGGTCTGCCTCCG; *Cdkn1a* (p21^Cip1^) reverse: CGGTCCCGTGGACAGTGAGCAG; *Cdkn2a* (p16^INK4a^) forward: CCCAACGCCCCGAACT; *Cdkn2a* (p16^INK4a^) reverse: GCAGAAGAGCTGCTACGTGAA; *Tnf* (TNF) forward: CTATGTCTCAGCCTCTTCTC; *Tnf* (TNF) reverse: CATTTGGGAACTTCTCATCC; *Il6* (IL-6) forward: AAGAAATGATGGATGCTACC; *Il6* (IL-6) reverse: GAGTTTCTGTATCTCTCTGAAG; *IL-1α* forward: ACGGCTGAGTTTCAGTGAGACC; *IL-1α* reverse: CACTCTGGTAGGTGTAAGGTGC; *IL-1β* forward: TGGACCTTCCAGGATGAGGACA; *IL-1β* reverse: GTTCATCTCGGAGCCTGTAGTG; *Mcp-1* forward: GCTACAAGAGGATCACCAGCAG; *Mcp-1* reverse: GTCTGGACCCATTCCTTCTTGG; *Pai-1* forward: CCTCTTCCACAAGTCTGATGGC; *Pai-1* reverse: GCAGTTCCACAACGTCATACTCG; *Cxcl-1* forward: TCCAGAGCTTGAAGGTGTTGCC; *Cxcl-1* reverse: AACCAAGGGAGCTTCAGGGTCA; *Cxcl-2* forward: CATCCAGAGCTTGAGTGTGACG; *Cxcl-2* reverse: GGCTTCAGGGTCAAGGCAAACT; *COX2* forward: GCGACATACTCAAGCAGGAGCA; *COX2* reverse: AGTGGTAACCGCTCAGGTGTTG; *iNOS* forward: GAGACAGGGAAGTCTGAAGCAC; *iNOS* reverse: CCAGCAGTAGTTGCTCCTCTTC; *p53* forward: CTCTCCCCCGCAAAAGAAAAA; *p53* reverse: CGGAACATCTCGAAGCGTTTA; *p19* forward: GGAGCTGGTGCATCCTGACGC; *p19* reverse: TGGCACCTTGCTTCAGGAGCTC; *Mmp3* forward: CTCTGGAACCTGAGACATCACC; *Mmp3* reverse: AGGAGTCCTGAGAGATTTGCGC; *Mmp12* forward: CACACTTCCCAGGAATCAAGCC; *Mmp12* reverse: TTTGGTGACACGACGGAACAGG; *Actb* (β-actin) forward: GATGTATGAAGGCTTTGGTC; *Actb* (β-actin) reverse: TGTGCACTTTTATTGGTCTC.

### Immunofluorescent staining

Primary MEFs were seeded into 8-well chamber slides and allowed to attach overnight at 20% oxygen. Cells were then fixed with 4% paraformaldehyde (PFA) for 10 min, permeabilized with 0.3% Triton X-100 in PBS for 10 min and blocked with 3% BSA in PBST for 1 hr in room temperature. Primary antibody incubation was performed at 4°C overnight and secondary antibody incubation for 1 hr at room temperature. Primary antibodies used are as follows: anti-p65 (CST #8242) and anti-NEMO (Santa Cruz sc-8330). Cell nuclei were counterstained with Vectashield mounting medium with DAPI. Five images were acquired for each sample at 60x magnification using an Olympus confocal microscopy.

### Senescence-associated β-galactosidase (SA-βgal) staining *in vitro* and *in vivo*

Primary MEFs were seeded into 6-well plates at 3x10^4^ cells per well, allowed to attach overnight and then treated with either vehicle or KU-55933 at 10 μM for 72 hours. Fresh fat tissues were preserved in ice-cold PBS prior to staining. MEFs and adipose tissues were then fixed in 2% formaldehyde and 0.2% glutaraldehyde in PBS for 10 minutes followed by incubation with SA-βgal staining solution (MEFs: pH 5.8; Fat: pH 6.0; 40 μM citric acid in sodium phosphate buffer, 5 μM K_4_[Fe(CN)_6_] 3H_2_O, 5 μM K_3_[Fe(CN)_6_], 150 μM sodium chloride, 2 μM magnesium chloride and 1 mg/ml X-gal dissolved in N, N-dimethylformamide) for 16-20 hours in a 37°C incubator without CO_2_ injector. To quantify, ten images were acquired randomly using a bright-field microscopy at 20x maginification. Total number of SA-βgal^+^ cells was normalized to the total cell number (DAPI) to obtain the percentage of SA-βgal^+^ cells.

### Health evaluation

Health assessments were conducted weekly to assess the age at onset and severity of numerous age-related conditions characteristic of Ercc1^-/∆^ mice, including dystonia, ataxia, kyphosis, tremor, muscle wasting, spontaneous activity and coat condition. In addition, body weight and grip strength were measured. All aging symptoms were scored on a scale of 0, 0.5 and 1, except for dystonia on a scale from 0 to 5. The sum of aging scores was used to determine the overall health of the individual animals, then averaged by genotype and age group.

### MDSPC isolation

The *Atm^+/-^*, *Ercc1^-/∆^Atm^+/-^* and *Ercc1^-/∆^* mice were sacrificed at 16 to 18 weeks of age, and MDSPC isolation was performed via a modified preplate technique as previously described [78, 79]. Briefly, the skeletal muscle tissue was minced and processed through a series of enzymatic dissociations: 0.2% of collagenase type XI (C7657, Sigma-Aldrich) for 1 hour, 2.4 units/ml of dispase (17105-041, Invitrogen) for 45 minutes, and 0.1% of trypsin-EDTA (15400-054, Invitrogen) for 30 minutes at 37°C. After enzymatic dissociation, the muscle cells were centrifuged and resuspended in proliferation medium (Dulbecco’s modified Eagle’s medium (DMEM, 11995-073, Invitrogen) supplemented with 10% fetal bovine serum (FBS, 10437-028, Invitrogen), 10% horse serum (HS, 26050-088, Invitrogen), 0.5% chicken embryo extract (CEE, CE650T-10, Accurate Chemical Co.), and 1% penicillin-streptomycin (15140-122, Invitrogen). The cells were then plated on collagen type I (C9791, Sigma-Aldrich) coated flasks. Different populations of muscle-derived cells were isolated based on their different adhesion characteristics. After 7 days, late preplate populations (slow-adhering cells), which have previously been described to contain the MDSPC fraction of cells, were obtained and cultured in proliferation medium [80].

### MDSPC proliferation assay

MDSPCs were cultured at 5,000 cells per well in collagen-coated 24-well plates in proliferation medium. Cell proliferation was tested with an MTS assay. After 3 days, proliferation medium was removed from wells and 100 μl of fresh proliferation medium was added to each well and allowed to equilibrate for 1 hr. Then, 20 μl of MTS reagent (Promega, Cat# G3582) was added to each well and incubated for 4 hrs. The optical density at 490 nm was measured using a spectrophotometer.

### Myogenic differentiation assay and fast myosin heavy chain staining

The cells were plated on 24 well plates (30,000 cells/well) in differentiation medium (DMEM supplemented with 2% FBS). Three days after plating, immunocytochemical staining for fast myosin heavy chain (MyHCf) was performed. Cells were fixed for 2 minutes in cold methanol (-20°C), blocked with 10% donkey serum (017-000-121, Jackson ImmunoResearch) for 1 hour and then incubated with a mouse anti-MyHCf (M4276, 1:250; Sigma-Aldrich) antibody for 2 hours at RT. The primary antibody was detected with an Alexa 594-conjugated anti-mouse IgG antibody (A21203, 1:500; Molecular probes) for 30 minutes. The nuclei were revealed by 4, 6-diamidino-2- phenylindole (DAPI, D9542, 100ng/ml, Sigma-Aldrich) staining. The percentage of differentiated myotubes was quantified as the number of nuclei in MyHCf positive myotubes relative to the total number of nuclei.

### Histologic analysis

Tissues were fixed in 10% neutral buffered formalin (NBF) overnight before embedding in paraffin. 5 μm sections were acquired using a microtome. Hematoxylin and eosin (H&E) staining was conducted following a standard protocol.

### Glycosaminoglycan (GAG) analysis

Snap frozen lumber spines were harvested at 12 weeks of age from Ercc1^-/Δ^ and Ercc1^-/∆^Atm^+/-^ mice. Nucleus pulposus (NP) tissue was isolated and dissected under a microscope and six lumbar intervertebral discs were pooled for analysis. GAG was isolated by papain digestion at 60°C for 2 hrs. Concentration of GAG was measured according to the 1,9-dimethymethylene blue (DMMB) procedure using chondroitin-6-sulfate (Sigma C-8529) as the standard. DNA concentration was measured using Pico Green assay (Molecular Probes). Fold change of GAG content was calculated by normalizing GAG to DNA concentration.

### Micro-computed tomography

Micro-computed tomography (μCT) of spines isolated from 12-week-old wild type (WT), *Ercc1^–/Δ^* and *Ercc1^–/Δ^Atm^+/-^* mouse littermates were acquired using the VivaCT 40 scanner (Scanco, Switzerland) with settings: energy 55 kvP, intensity 145 μA, integration time 200ms, isotropic voxel size 15 μm, threshold 235. 3D morphometric analysis were performed on lumbar vertebrae segments (L3-S1).

### Statistical analysis

All values were presented as mean+/-S.E.M. Microsoft Excel and Graphpad Prism 6 were used for statistical analysis. Two-tailed Student’s t-test was performed to determine differences between two groups. A value of p <0.05 was considered as statistically significant, shown as *p <0.05, **p <0.01, and ***p < 0.001.

## Supplementary Material

Supplementary Figures
