# Supplementary material for: ATM is a key driver of NF-κB-dependent DNA-damage-induced senescence, stem cell dysfunction and aging
Source: Aging (Albany NY). 2020 Mar 22;12(6):4688–710. doi: 10.18632/aging.102863 (PMC7138542; doi:10.18632/aging.102863)
Supplement: Supplementary Figures [file aging-12-102863-s001..pdf]

SUPPLEMENTARY FIGURES

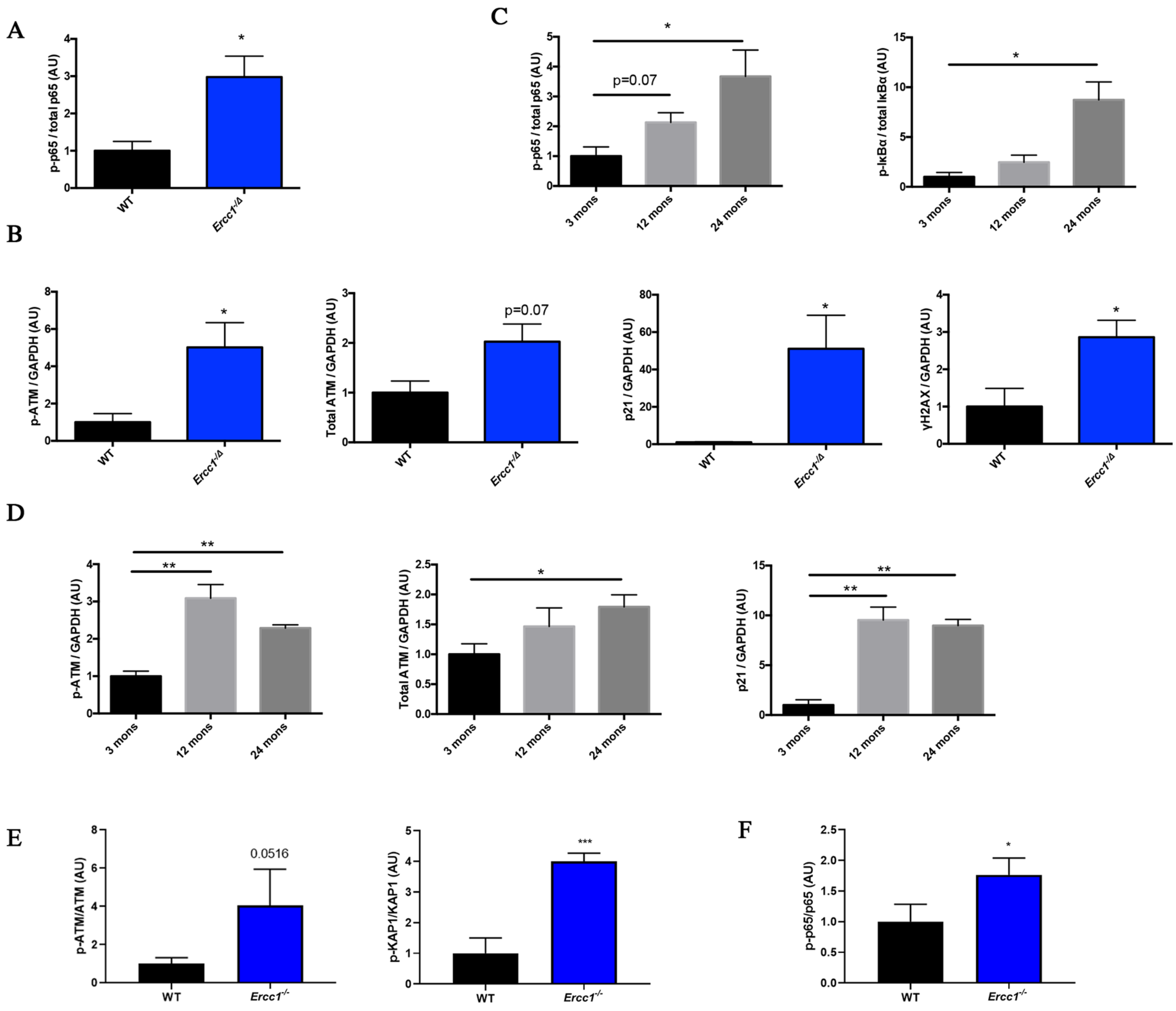

Supplementary Figure 1. DDR and NF-κB are activated in tissues from progeroid *Ercc1*$^{-/\Delta}$ and old WT mice. Densitometric analysis of Western Blot shown in Figure 1A (**A**), Figure 1B (**B**), Figure 1C. (**C**) and Figure 1D (**D**). (**A**–**B**) Two-tailed Student's t-test was performed. (**C**–**D**) One-way ANOVA was performed. *p <0.05, **p <0.01 or as indicated in the figure.

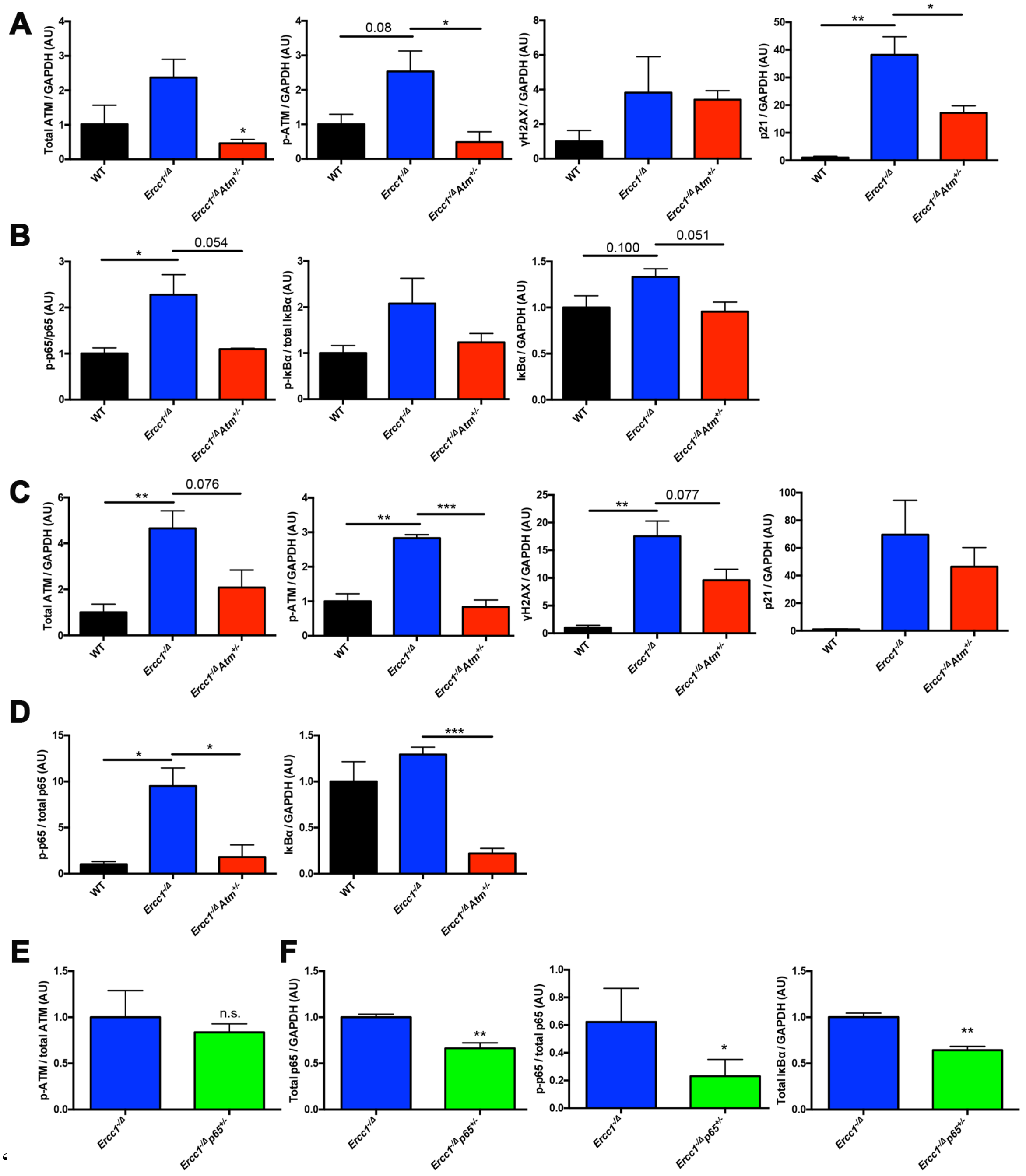

Supplementary Figure 2. Atm haploinsufficiency reduces senescence by regulating NF-κB activation. Densitometric analysis of Western Blot shown in Figure 7A (**A**), Figure 7B (**B**), Figure 7C (**C**), Figure 7D (**D**), Figure 7E (**E**) and Figure 7F (**F**). P values were determined using a Student's t-test. *p <0.05, **p <0.01, ***p<0.001 or as indicated in the figure.

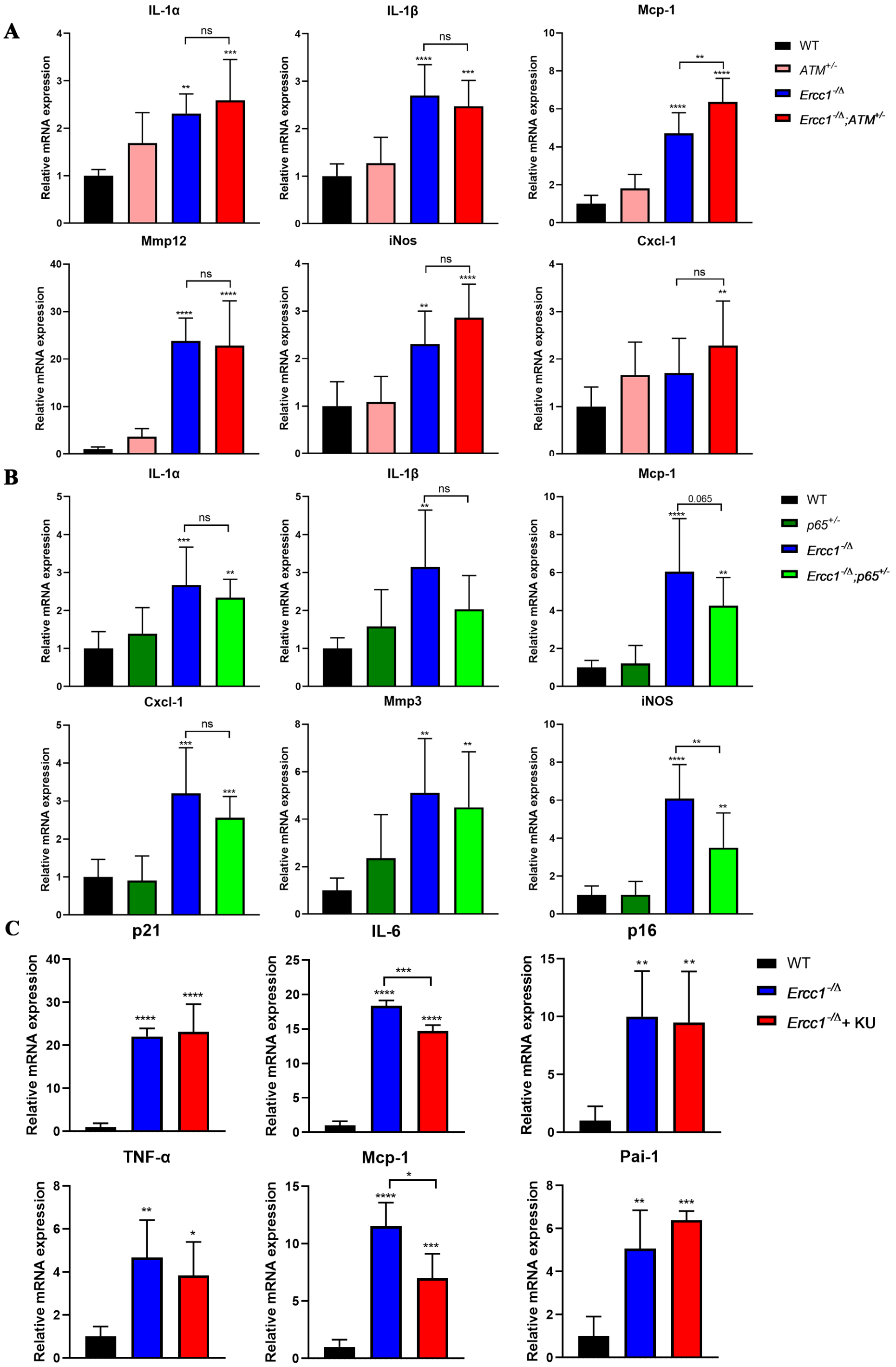

Supplementary Figure 3. Genetic reduction or pharmacologic inhibition of *Atm* or *NF-κB* attenuates aging phenotypes and reduces cellular senescence *in vivo*. (**A**) qRT-PCR analysis of mRNA expression in liver from 12-week-old WT, *Atm*$^{+/-}$, *Ercc1*$^{-/\Delta}$ and *Ercc1*$^{-/\Delta}$*Atm*$^{+/-}$ mice. n=3-6 per group. (**B**) qRT-PCR analysis of mRNA expression in liver from 10 to 12-week-old WT, *p65*$^{+/-}$, *Ercc1*$^{-/\Delta}$ and *Ercc1*$^{-/\Delta}$p65$^{+/-}$ mice. n=4-5 per group. (**C**) mRNA expression of senescence markers in the kidney of 12-week-old *Ercc1*$^{-/\Delta}$ mice treated with 10 mg/kg of KU-55933 intraperitoneally 3 times per week for two weeks. n = 3 per group. Graphs represent mean+/- s.e.m. P value was determined using Student's t-test. *p<0.05, **p<0.01, ***p <0.001, ****p <0.0001.
